# Comparison of Survival Rates of Stainless-Steel Crowns Placed with and without Pulpotomy: A Two-Year Retrospective Study

**DOI:** 10.1155/2020/8883189

**Published:** 2020-10-22

**Authors:** Fatmah N. AlMotawah, Sharat Chandra Pani, Tala AlKharashi, Saleh AlKhalaf, Mohammed AlKhathlan, Fahad AlSultan, Ahmad AlMughirah

**Affiliations:** ^1^Pediatric Dentistry, Riyadh Elm University, Riyadh, Saudi Arabia; ^2^Division of Paediatric Dentistry, Schulich School of Medicine and Dentistry, University of Western Ontario, London, ON, Canada; ^3^College of Dentistry, King Saud Bin Abdulaziz University for Health Sciences, Riyadh, Saudi Arabia; ^4^Private Dental Practice, AlAhsa, Saudi Arabia; ^5^College of Dentistry, Prince Sattam Bin Abdulaziz University, AlKharj, Saudi Arabia

## Abstract

**Aim:**

This study aimed to retrospectively compare the survival outcomes over two years between teeth with proximal dental caries that were restored with stainless-steel crowns to those that were pulpotomized and then restored with a stainless-steel crown in patients who were rehabilitated under general anesthesia. *Participants and Methods*. The records of 131 patients aged between two to six years who had stainless-steel crowns placed under general anesthesia and had two-year follow-up were screened. 340 teeth with moderate proximal caries on the radiograph (D2) were included in the study. Of these, 164 teeth were treated with a pulpotomy and stainless-steel crown, while 176 teeth were crowned without a pulpotomy. The type of each tooth was compared using the Chi-squared test and Kaplan–Meier survival analysis, and curves were plotted based on the two-year outcomes.

**Results:**

Treatment: the sample comprised 59 males (mean age 4.73 years, SD ± 1.4 years) and 72 females (mean age 5.2 years, SD ± 2.0 years). The Kaplan–Meier regression model showed no significant difference in survival outcomes between teeth that had been pulpotomized and those that had not (*p* = 0.283).

**Conclusion:**

Within the limitations of the current study, we can conclude that performing a pulpotomy does not influence the survival outcome of mild/moderate proximal caries restored with stainless-steel crowns under general anesthesia.

## 1. Introduction

Dental caries is one of the most common and widespread pathological diseases worldwide affecting 2.43 billion people globally that is almost one-third of population [1]. Caries is a result of bacterial biofilm colonization on the tooth surface that utilizes carbohydrates of food and drinks into acids leading to low PH levels in the oral cavity, which cause enamel demineralization, followed by enamel and dentin caries [2]. Moreover, with constant high intake of carbohydrate and sugar in addition to low-fluoride exposure, a distraction can cause to a tooth structure inducing pain and cavities, leading to pulpitis, tooth loss, and infection in late stages [3–5]. Early childhood caries (ECC), defined as the presence of one or more dental caries lesion in 71 months of age or younger, has been recognized as a global challenge to pediatric oral health [6]. Recent data shows that ECC continues to remain a serious public health challenge in Saudi Arabia with close to 80% of children below the age of 6 years affected by early childhood caries [7].

The management of early childhood caries in children is often carried out under general anesthesia [8, 9]. The management of proximal caries in primary teeth is a controversial topic [10, 11]. While there is consensus that the restoration of choice for a primary tooth with multiple carious surfaces is a stainless-steel crown, there is no consensus on pulp therapy for proximal caries that does not reach the pulp on a radiograph [11]. While some authors have recommended that all teeth with a marginal ridge destroyed by dental caries should be pulp-treated [12], others have suggested that covering teeth with a stainless-steel crown without a pulpotomy would be just as effective [8, 10, 11]. Investigators have cited the success of the use of the “Hall Technique” to support the concept of minimal intervention [10].

The treatment of children under general anesthesia raises certain concerns since a failure of the treatment often means that the retreatment would need to be carried out under general anesthesia as well [8, 12, 13]. Pulpotomies performed under general anesthesia have been shown to have a failure rate of 1.1% to 3% [14, 15]. While the failure of SSCs placed under GA has been reported to be as high 5.6%, it has been suggested that the failure of the SSC is often caused due to the failure of the pulp therapy [12, 15]. Despite being the restoration for deep caries and after pulp treatment, there is little data on the success or failure of proximal carious lesions restored under general anesthesia without pulp therapy.

Given this lack of data, the aim of this study was to retrospectively compare the survival outcomes over a two-year period between teeth with proximal dental caries that were restored with stainless-steel crowns to those that were pulpotomized and then restored with stainless-steel crowns in patients who were rehabilitated under general anesthesia.

## 2. Methods

### 2.1. Ethical Approval

Ethical approval was obtained from the Institutional review board of the Riyadh Elm University (RC/IRB/2019/349). The deidentified patient records were obtained from the clinical record manager of the university. There was no patient participation in the study, and consent to view deidentified records had been obtained from the patients by the hospital at the time of the operation.

### 2.2. Study Design

A retrospective cohort study was used whereby the records of the admitted patients and their follow-up records were utilized to obtain the data.

### 2.3. Inclusion Criteria

The records of patients aged between 2 and 8 who had undergone the placement of at least one stainless-steel crown under general anesthesia were included in the study. The teeth had received stainless-steel crowns for the management of proximal caries, which was close to, but not approaching the pulp on a radiograph (ADA classification D2). The only healthy children as classified by the American Society for Anesthesiology (ASA) as ASA1 were included in the study.

### 2.4. Sample Studied

**Figure 1:**
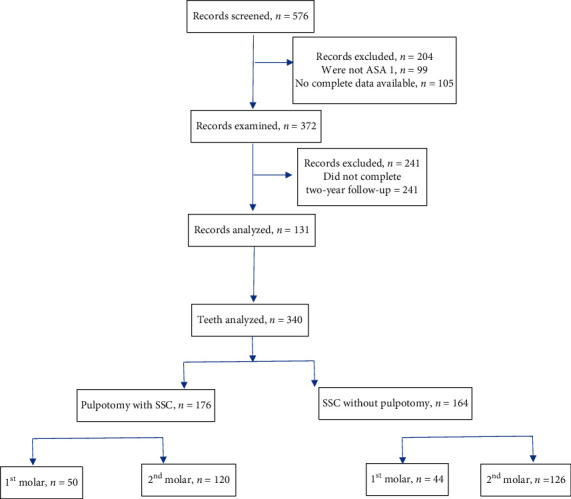
Distribution of the sample.

A total of 576 records of patients undergoing pediatric dental rehabilitation under general anesthesia from 2012 to 2018 were screened. The files which did not have complete data (*n* = 105) and those who were not ASA 1 (*n* = 99) were excluded from the study. The remaining 372 records were screened to determine those who had completed two years of continuous six month recall visits (Figure 1). Of the 131 records (59 males and 72 females) that met the selection criteria, there were 340 teeth that met the inclusion criteria (caries = D2). Of these, 176 of these teeth were treated with pulpotomy and followed with stainless-steel crown (SSC) (1^st^ molars = 50, 2^nd^ molars = 120), while 164 teeth were only treated with SSC without pulpotomy (1^st^ molars = 44 and 2^nd^ molars = 126). A post hoc sample power calculation was performed using the G-power sample power calculator (Universtat Kiel, Kiel, Germany). For a Kaplan–Meier regression model, assuming an effect size of 0.15, and alpha of 0.05, the sample of 340 teeth yielded a power of 0.978.

### 2.5. Outcome Measures

The outcome measures recorded included the presence or absence of pulpotomy, success or failure of the SSC, and/or pulpotomy. The other variables recorded included the age of the child, the gender of the child, and the type of tooth.

### 2.6. Statistical Methods

Patient age between the genders was measured using the independent *t*-test. The type of each tooth was compared using the Chi-square test. Kaplan–Meier prediction curve was used to assess the survival times of the treatment. A binomial regression model with the survival outcome as dependent variables and type of treatment (with or without pulpotomy) and type of tooth as cofactors was designed. All statistical analysis was done using SPSS version 23 (IBM-SPSS, Armonk NY, USA), and the level of confidence was set at *p* < 0.05 for all tests.

## 3. Results

The sample comprised 340 teeth from 131 patients who completed a two-year follow-up of their treatment. The sample comprised 59 males (mean age 4.73 years, SD ± 1.4 years) and 72 females (mean age 5.2 years, SD ± 2.0 years). There was no statistical difference in the age of the males and females (*t* = −1.469, *p* = 0.145).

**Figure 2:**
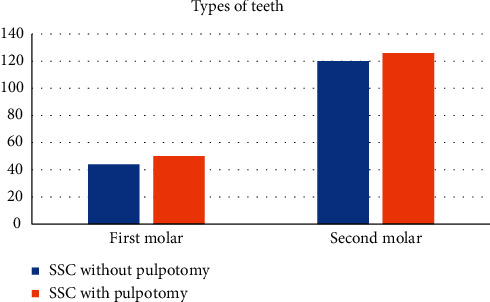
Types of teeth and their distribution among the groups.

Of the 340 teeth analyzed, there were 164 teeth that underwent pulpotomy and 176 teeth that were crowned without a pulpotomy (Figure 2). Although the numbers of each type of tooth were similar in both groups (Chi-square = 0.106, *p* = 0.809), there were significantly more second primary molars than first primary molars (Chi-square = 67.95, *p* < 0.001).

**Figure 3:**
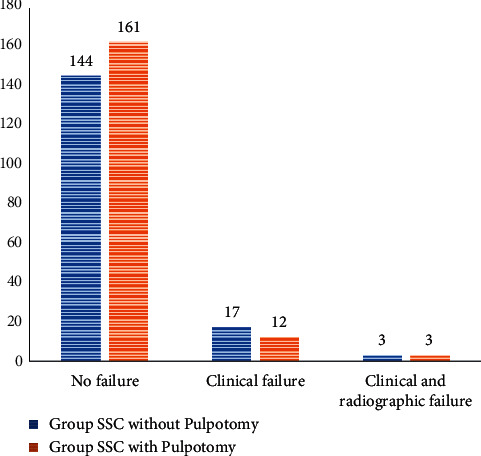
Types of failure in the two groups.

When the total number of failures was analyzed, it was observed that 29 of the 340 teeth analyzed had patients reporting with a history of clinical failure and 6 reported with both clinical and radiographic failure (Figure 3). When these were compared between the group with pulpotomy and those without pulpotomy, it was observed that the group without pulpotomy had higher numbers of clinical failures (*n* = 17) than their counterparts with pulpotomy (*n* = 12). However, these differences were not statistically significant (Chi-square = 1.388, *p* = 0.499).

**Figure 4:**
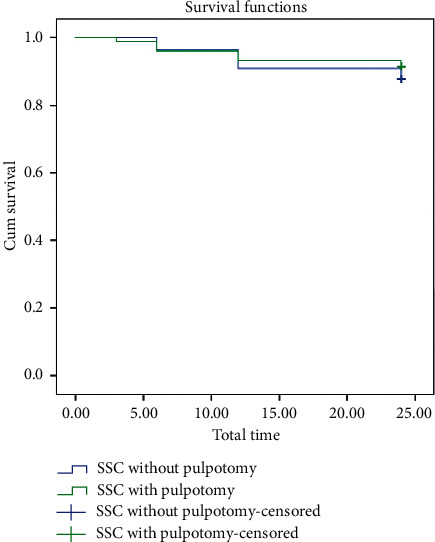
Kaplan–Meier survival plot for stainless-steel crowns with and without pulpotomy.

When the Kaplan–Meier survival analysis was performed for the two groups, it was observed that the group with the pulpotomy had a mean survival time of 22.68 months, while the group without pulpotomy had a mean survival time of 22.90 months (Figure 4). However, the analysis showed that the difference was not statistically significant (*p* = 0.283) (Table 1).

When a binomial regression was performed with the outcome of the tooth as the dependent factor and the type tooth (first or second molar) and the presence or absence of pulpotomy as dependent variables, it was observed that neither the presence of a pulpotomy (*p* = 0.675) nor type of tooth (*p* = 0.982) had a significant impact on the outcome (success or failure) of the tooth (Table 2).

## 4. Discussion

The management of proximal caries under general anesthesia has been a topic of interest to pediatric dentists across the world [9, 13, 14, 16]. Traditionally, the SSC has been viewed as an aggressive form of treating dental caries in primary molars [12, 16]. This meant that the SSC, especially when performed under GA, has been combined with a pulpotomy or pulpectomy [8, 12]. However, with advent of the Hall technique, there has been an increased recognition of the SSC as a form of minimal intervention dentistry [11]. This study aimed to evaluate if placing a pulpotomy on teeth with no history of spontaneous pain and proximal dental caries improved the survival outcomes for SSCs placed under GA.

There has been a change in the way we measure and record dental caries with systems such as the ICDAS and the ADA classification seeking to provide a more sensitivity than the conventional dft or dfs for primary teeth [17]. Our decision to use the ADA classification was based on the fact that it uses radiographic depth of the lesion as a measure of the severity of the dental caries [18]. This allowed for the use of radiographic records to assess secondary data. The decision to use D2 caries that was more than one-third into dentin but less than two thirds into dentin was based on the fact that such lesions have been documented to be subjected to “intentional” pulpotomies [12, 14, 15].

Our sample comprised significantly more second primary molars than first primary molars. This is despite that fact that most studies show the first primary molar to be more commonly affected with dental caries than the second primary molar [19, 20]. One possible explanation for this could be the fact that, given the advanced state of dental caries in these teeth, first primary molars are often extracted when the child is treated under general anesthesia.

The overall failure rate of SSCs placed in our study was higher than previously reported in literature, with most of the failures occurring due to debonding of the crown. One reason for this was that most studies on the success of stainless-steel crowns do not consider the debonding of the crown as a “true failure” [15, 21].

Although there was no significant impact of performing a pulpotomy, it is interesting to note that the teeth that were restored without pulp therapy actually showed a longer projected survival time. This seems to lend credence to recent work on the placement of SSCs chair-side that has shown that if there is appropriate case selection, the survival of teeth with proximal caries is not affected by the performance of a pulpotomy [10, 11].

There are many investigators who argue that given the invasive nature of treatment under general anesthesia makes the use of pulp therapy in proximal caries lesions the standard of care [12, 15]. The rates of failure of restorations following general anesthesia, however, seem to suggest that there is a risk of failure even after pulpectomies were performed [8, 12, 15]. Studies from across the globe have shown that while general anesthesia improves the oral health-related quality of life of children, the most significant predictor of success is the implementation of good postoperative oral hygiene and parental oral health promotion [8, 22–25].

The results of the study must be viewed keeping in mind that only a specific type of proximal carious lesion was studied. The retrospective nature of the study also meant that severity of dental caries was only determined radiographically and there was no way of confirming the clinical nature of the lesion. Furthermore, the lesions were not matched for patient, and therefore, it was not possible to know the effects of individual patient factors such as diet, oral hygiene, or parental motivation. Despite these limitations, the results of the study suggest the need for future prospective studies to investigate these factors. There is also a need for future studies to examine the possibilities of innovative esthetic alternatives to stainless-steel crowns such as 3-D printing and CAD-CAM to create affordable and esthetic posterior restorations [26, 27].

## 5. Conclusion

Within the limitations of the study, we can conclude that pulp therapy does not significantly influence the survival of moderate proximal caries lesions restored with stainless-steel crowns under general anesthesia.

## Figures and Tables

**Table 1:** Survival analysis for stainless-steel crowns with and without pulpotomy.

Group	Mean				Sig
	Estimate	Std. error	95% confidence interval		
			Lower bound	Upper bound	
SSC without pulpotomy	22.683	0.340	22.016	23.350	0.283
SSC with pulpotomy	22.909	0.323	22.276	23.542	
Overall	22.800	0.231	22.348	23.252	

**Table 2:** Binomial logistic regression showing the association of the type of tooth and presence of pulpotomy to the survival outcome of the tooth.

Variables in the equation		B	S. E.	Wald	Df	Sig.	Exp (B)
Step 1^a^	Presence of pulpotomy	−0.393	0.361	1.185	1	0.276	0.675
	Type of tooth	−0.018	0.018	1.064	1	0.302	0.982
	Constant	−0.687	1.258	0.298	1	0.585	0.503

^a^Variable(s) entered on step 1: type of tooth, presence of pulpotomy. Dependant variable: survival outcome.

## Data Availability

Data will be made available upon reasonable request to the corresponding author.
